# Reproductive and Perinatal Outcomes of Embryo Transfer in Elite Hanwoo Cows

**DOI:** 10.1002/vms3.70828

**Published:** 2026-02-15

**Authors:** Mi‐Ryung Park, Se Young Lee, Sang‐Rae Cho, Seungmin Ha, Yeoung‐Gyu Ko

**Affiliations:** ^1^ Animal Genetic Resources Research Center National Institute of Animal Science, Rural Development Administration Gyeongnam South Korea

**Keywords:** calving success, dystocia, Elite Hanwoo, embryo transfer, MOET, OPU‐IVF

## Abstract

Veterinarians should be vigilant in limiting embryonic, foetal and neonatal losses through appropriate reproductive programs and management strategies following natural mating and artificial insemination, especially after embryo transfer (ET) procedures, due to their high associated costs in Elite Hanwoo breeding programs. To establish a breeding population of Hanwoo cows with top 1% genomic estimated breeding values (GEBVs) for carcass weight and compare reproductive outcomes between OPU‐IVF and MOET. Elite donor cows were assigned to either OPU‐IVF or MOET groups, and the resulting embryos were transferred to synchronized recipient cows. Pregnancy rate, calving success, abortion, birth weight, sex ratio and dystocia were evaluated. The MOET group showed 100% calving success with no abortions; the OPU group had a 68.2% calving rate and 31.8% abortion rate. The sex ratio of the resulting offspring was similar between the groups, with female calves accounting for 37.5%–40.0% and male calves for 60.0%–62.5% of total calves. While the mean birth weights of female calves did not significantly differ between the groups (32.0–32.8 kg), male calves from the OPU group exhibited a significantly higher average birth weight (36.9 kg) than those from the MOET group, with a mean difference of 4.9 kg (*p* < 0.05). Spontaneous calving was more frequent in MOET (62.5%), while induced parturition was common in OPU (56.7%). Dystocia incidence was higher in OPU, especially with early induction. OPU‐IVF was linked to higher abortion and dystocia risks, while MOET showed more favourable calving outcomes. Appropriate embryo production strategy and careful periparturient management are crucial for successful Hanwoo reproduction.

## Graphical abstract

Reproductive outcomes following embryo transfer in Elite Hanwoo cows differed by embryo production method. OPU‐IVF was associated with higher abortion, increased birth weight of male calves and greater dystocia risk, whereas MOET resulted in more favourable calving outcomes, highlighting the importance of embryo production strategy and periparturient management.

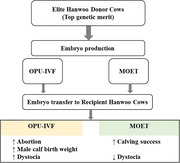

## 1 Introduction

Hanwoo (*Bos taurus coreanae*) is a native Korean cow breed with high cultural and economic value (S. H. Lee et al. 2014; Jo et al. 2012). Hanwoo cows are well recognized for their intramuscular fat (marbling), which contributes to their classification as a premium beef breed. This superior marbling quality enhances both tenderness and flavour, making Hanwoo beef highly desirable among consumers (Gajaweera et al. 2018; B. Lee and Choi 2019). Despite these advantages in terms of meat quality, Hanwoo cows exhibit relatively lower reproductive performance, with a mean age at first calving of approximately 24 months and a calving interval of about 399 days, compared with the commonly targeted calving interval of ≤ 365 days in beef cattle production systems. To address this issue, assisted reproductive technologies such as artificial insemination (AI) and embryo transfer (ET) have been widely implemented (Kang et al. 2024; Park et al. 2023).

Genetically superior and economically valuable Hanwoo cows, referred to as Elite Hanwoo cows, serve as core genetic resources in national breeding programs. Elite Hanwoo cows are defined as individuals with traceable pedigree records and proven carcass performance, including progeny with a marbling score of 1++ (scores 8 or 9), a yield grade of B or higher, a carcass weight of ≥ 480 kg, a ribeye area of ≥ 110 cm^2^, visual appraisal scores of ≥ 80 and confirmed parentage via DNA testing (Korea Animal Improvement Association 2021a). These elite animals are selected based on national genetic evaluations and progeny performance testing, and their superior traits are propagated using AI and ET technologies. As a result, the establishment of high‐performance donor cow herds and ET practices has become increasingly common, accompanied by a growing demand for Elite Hanwoo cow‐derived embryos. Official records show a notable increase in issued certificates for embryos derived via ovum pick‐up (OPU), from 7947 cases in 2016 to 20,485 cases in 2021 (Korea Animal Improvement Association 2021b).

OPU and multiple ovulation and ET (MOET) are reproductive technologies employed to collect oocytes from livestock such as cattle, sheep and goats. These methods allow for non‐surgical collection of oocytes, which are subsequently used for embryo production through in vivo fertilization (MOET) or in vitro fertilization (OPU‐IVF). These technologies are particularly valuable for propagating the genetics of superior animals and are widely applied in genetic and reproductive research (Ferré et al. 2023; Márquez‐Moya et al. 2025; Pimentel et al. 2012; Toosi et al. 2009). Induced parturition is a common practice in cattle during late gestation, when natural calving is delayed or when risks to the dam or foetus are anticipated. This process involves the administration of agents such as prostaglandins, oxytocin and corticosteroids to artificially initiate labour (Kang et al. 2019; Wieczorek et al. 2020). Accordingly, the primary objective of reproductive management is to ensure the health and safety of dams and neonates by promoting timely calving.

Although Elite Hanwoo cows are produced across various regions of South Korea, there is a lack of foundational data regarding their genetics, reproduction, disease susceptibility and growth characteristics. Moreover, some farms that maintain Elite Hanwoo cow herds have reported challenges such as incidences of dystocia, stillbirths and even dam mortality following ET. These issues underscore the need for systematic data collection and management strategies.

In conclusion, the present study was conducted to generate baseline data related to induced parturition following ET in Elite Hanwoo cows produced via OPU or MOET. Specifically, the aim of this study was to examine labour duration, incidence of dystocia and birth weight of new‐born calves. Our study could support the expansion of Elite Hanwoo cow foundation herds, alleviate the challenges faced by producers and contribute to the overall quality improvement in the Hanwoo cow industry.

## 2 Materials and Methods

### 2.1 Animals

The experiment was conducted using Elite Hanwoo cows with a carcass genomic breeding value (CGBV) ranking within the top 0.02%–0.56%. The cows were aged between 54 and 74 months, with body weights ranging from 720 to 856 kg. These cows were selected from a reference population of 18,000 individuals, among which the top 1% in terms of CGBV were considered. The selection criteria included external evaluation, genetic potential and progeny information scores of 80 or higher, as assessed by the Hanwoo Improvement Center. The experiments adhered to the animal experiment ethics guidelines (approval no. NIAS2023‐0609) of the Animal Genetic Resources Research Center of the Rural Development Administration (RDA).

### 2.2 Oestrous Synchronization, AI, Blastocysts Collection and ET

**FIGURE 1:**
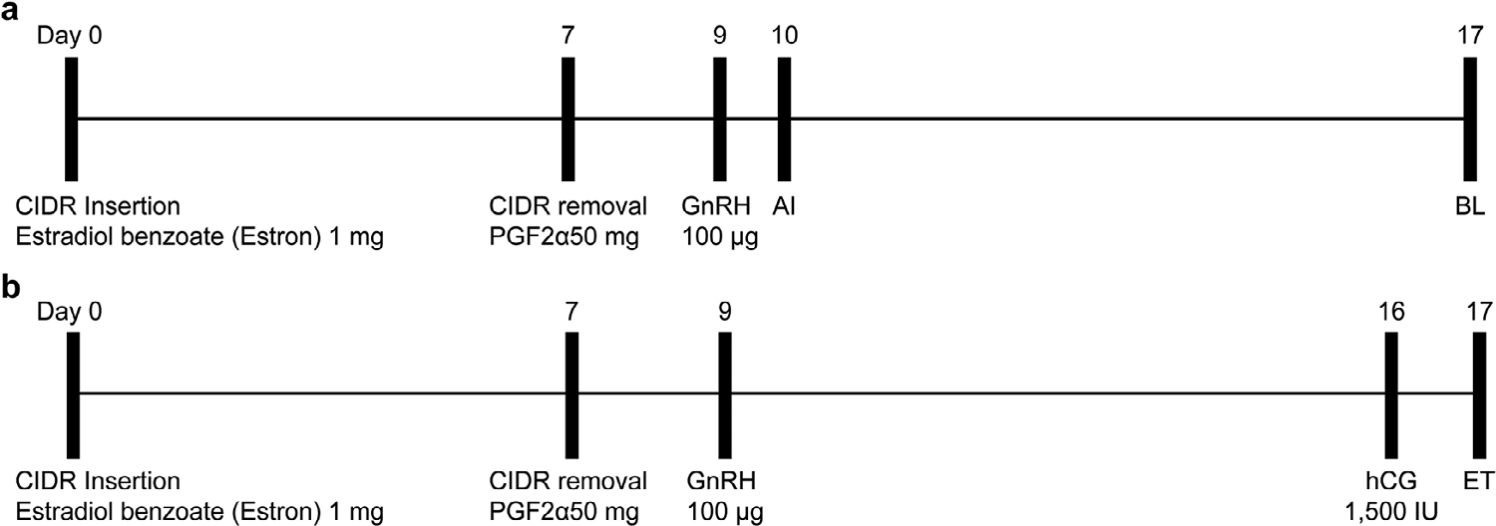
Oestrus synchronization schedule of recipient and donor cow preparation. (a) Oestrous synchronization schedule for blastocyst collection in the donor group. (b) Oestrous synchronization schedule for the recipient group.

Oestrous synchronization of donor and recipient Hanwoo cows was performed based on previously established protocols, as illustrated in Figure 1 (Bó and Mapletoft 2014; Facioli et al. 2020; Garcia et al. 1992). For oestrus synchronization, only clinically healthy cows without pathological ovarian or uterine conditions were selected. In the donor group, a progesterone‐releasing intravaginal device (CIDR; InterAg, Hamilton, New Zealand) was inserted on Day 0, concurrently with an intramuscular injection of 1 mg estradiol benzoate (Estron; Samyang Anipharm, Seoul, Republic of Korea).

On Day 7, the CIDR was removed, and 25 mg of prostaglandin F_2_α (PGF_2_α, Lutalyse; Phamacia Co. Puurs, Belgium) was administered intramuscularly to induce luteolysis. On Day 9, 100 µg of gonadotropin‐releasing hormone (GnRH; Fertagyl; Intervet, Boxmeer, the Netherlands) was injected to synchronize the timing of ovulation. AI was performed 10 days after treatment initiation using frozen‐thawed semen from proven Hanwoo bulls, with two inseminations per cow at 12‐h intervals, each using one straw of semen. Blastocyst‐stage embryos were collected on Day 7 post‐insemination by nonsurgical uterine flushing, with both uterine horns flushed separately using approximately 500 mL of flushing medium per horn. (Figure 1a).

The recipient cows consisted of general Hanwoo cows, and the treatment protocol from CIDR insertion to GnRH administration was conducted in the same manner as that in the donor group. Seven days after GnRH injection, 1500 IU of hCG (Chorulon; MSD, the Netherlands) was administered intramuscularly. One day later, rectal palpation was performed to assess the corpus luteum (CL) grade, and the blastocysts were transferred into the uterine horn ipsilateral to the ovary, bearing a functional CL (Figure 1b).

### 2.3 OPU and In Vitro Embryo Production

The OPU procedures were conducted once weekly by the same experienced technician using transvaginal ultrasonography. Donor cows were restrained, and a 7.5‐MHz transducer was inserted transvaginally to visualize the ovaries. Follicles with diameter ranging from 2 to 10 mm were aspirated using an 18‐gauge needle. Follicular fluid was collected under negative pressure (−70 mmHg) into collection tubes containing phosphate‐buffered saline supplemented with heparin (10 IU/mL). In vitro embryo production was conducted following previously described protocols (S. Y. Lee et al. 2024). All chemicals and reagents were purchased from Sigma Chemical Co. (St. Louis, MO, USA), unless stated otherwise. Following transport to the laboratory, the aspirated follicular fluid was filtered (Sarstedt, Numbrecht, Germany) and cumulus‐oocyte complexes were selected under a stereomicroscope (Olympus Co., Tokyo, Japan). The retrieved oocytes were matured for 22 h in a maturation medium based on TCM‐199 supplemented with 10% foetal bovine serum (Gibco), 7 µg/mL follicle‐stimulating hormone (Vetoquinol, USA Inc.), 500 IU/mL human chorionic gonadotropin (hCG; Intervet International BV), 0.2 mM sodium pyruvate and 0.785 mM L‐cysteine. Matured oocytes were fertilized in vitro using frozen‐thawed semen from proven Hanwoo bulls, which was processed by Percoll density gradient centrifugation to select motile spermatozoa in Tyrode's albumin–lactate–pyruvate medium supplemented with 10 µg/mL heparin, 5 mM caffeine, 1.387 mM D‐glucose and 6 mg/mL bovine serum albumin for 6 h. Fertilization was carried out at a final sperm concentration of 1∼2 × 10^6^ sperm/mL. The presumptive zygotes were cultured in mCR1aa medium (MK Biotech, Daejeon, South Korea) for 7 days at 38°C in a controlled atmosphere of 5% CO_2_ and 5% O_2_ to promote blastocyst development. The produced blastocysts were evaluated based on the grading system recommended by the International Embryo Technology Society (IETS), and only Grade 1 and 2 blastocysts were selected for ET.

### 2.4 Pregnancy Diagnosis and Parturition Monitoring

Pregnancy diagnosis was initially performed 45 days after ET using a commercial pregnancy detection kit (Alertys Pregnancy Test; IDEXX, Westbrook, USA) that detects pregnancy‐associated glycoproteins (PAGs) in bovine blood. Further confirmation of pregnancy was conducted at 2, 4 and 6.5 months of gestation using rectal palpation and ultrasonography (Sonovet Pico, Medison, South Korea). For animals undergoing natural parturition, monitoring was conducted at 2‐h intervals during the 72 h before and after the expected calving date. The duration of labour was recorded from the onset of parturition (rupture of the amniotic sac) to its completion (expulsion of the foetus).

In cases of induced parturition, prostaglandin F_2_α (PGF_2_α) was administered to synchronize the timing of calving and to ensure appropriate periparturient management. Induction was performed at ≥ 284 days of gestation, taking into account the average gestation length of Hanwoo cattle (approximately 285 days), to avoid interference with physiologically normal calving. Cows were then assigned to one of three groups based on the timing of parturition relative to the expected calving date: 1 day before, on the expected date or 1 day after. Each group received an intramuscular injection of 5 mL prostaglandin F2α (PGF2α), and subsequent parturition was closely monitored. In cases of dystocia, as determined by a licensed veterinarian, interventions such as manual rope‐assisted traction and/or episiotomy were performed to ensure the safe delivery of the foetus.

### 2.5 Statistical Methods

Statistical analyses were performed using SAS Enterprise Guide 7.1 (SAS Institute Inc., Cary, NC, USA). Treatment differences were evaluated using a *t*‐test based on the least significant difference (LSD) method. Results with a *p*‐value of less than 0.05 were considered statistically significant. Data are presented as mean ± standard error (SE).

## 3 Results

### 3.1 Pregnancy and Calving Outcomes Between the MOET and OPU Groups

A total of three donor cows were enrolled in the MOET group, yielding 39 embryos (13 embryos per donor). In the OPU group, three donor cows underwent OPU procedures, resulting in 420 oocytes and 251 embryos following in vitro fertilization. After morphological classification, 16 blastocysts were obtained from MOET‐derived embryos and 97 blastocysts from OPU‐derived embryos. These blastocysts were transferred to 16 and 97 synchronized recipient cows, respectively. In the MOET group, 8 out of 16 recipients (50.0%) were confirmed to be pregnant, and all eight successfully maintained pregnancy and gave birth (100%). In the OPU group, embryos were transferred to 97 recipient cows, 44 of which were confirmed to be pregnant (45.4%). Among these, 30 recipients (68.2%) successfully delivered calves, whereas 14 (30.4%) experienced early pregnancy loss. Notably, 12 of the 14 cows (85.7%) miscarried at approximately 4 months of gestation. However, all recipients confirmed to be pregnant at 7 months of gestation successfully carried to term. The pregnancy and calving rates were higher in the MOET group than in the OPU group (Table 1).

**TABLE 1:** Pregnancy and calving rates following Elite Hanwoo cow embryo transfer.

	No. of recipients	%Pregnancy (head)	%Parturition (head)	%Stillbirth (head)
MOET	16	50.0 (8)	100.0 (8)	0
OPU	97	45.4 (44)	68.2 (30)	31.8 (14)^a^

^a^ Among the 14 cases of stillbirth, 12 were aborted during early pregnancy, whereas 2 were aborted in the late gestation period.

### 3.2 Sex Ratio and Birth Weight of Calves Between the MOET and OPU Groups

The sex ratio was similar between the MOET and OPU groups, with female proportions of 37.5% and 40.0% and male proportions of 60.0% and 62.5%, respectively (Table 2).

**TABLE 2:** Sex ratios of calves in the MOET and OPU groups.

	No. of calves	Sex ratio of calves (%)	
		Female	Male
MOET	8	3 (37.5)	5 (62.5)
OPU	30	12 (40.0)	18 (60.0)

**FIGURE 2:**
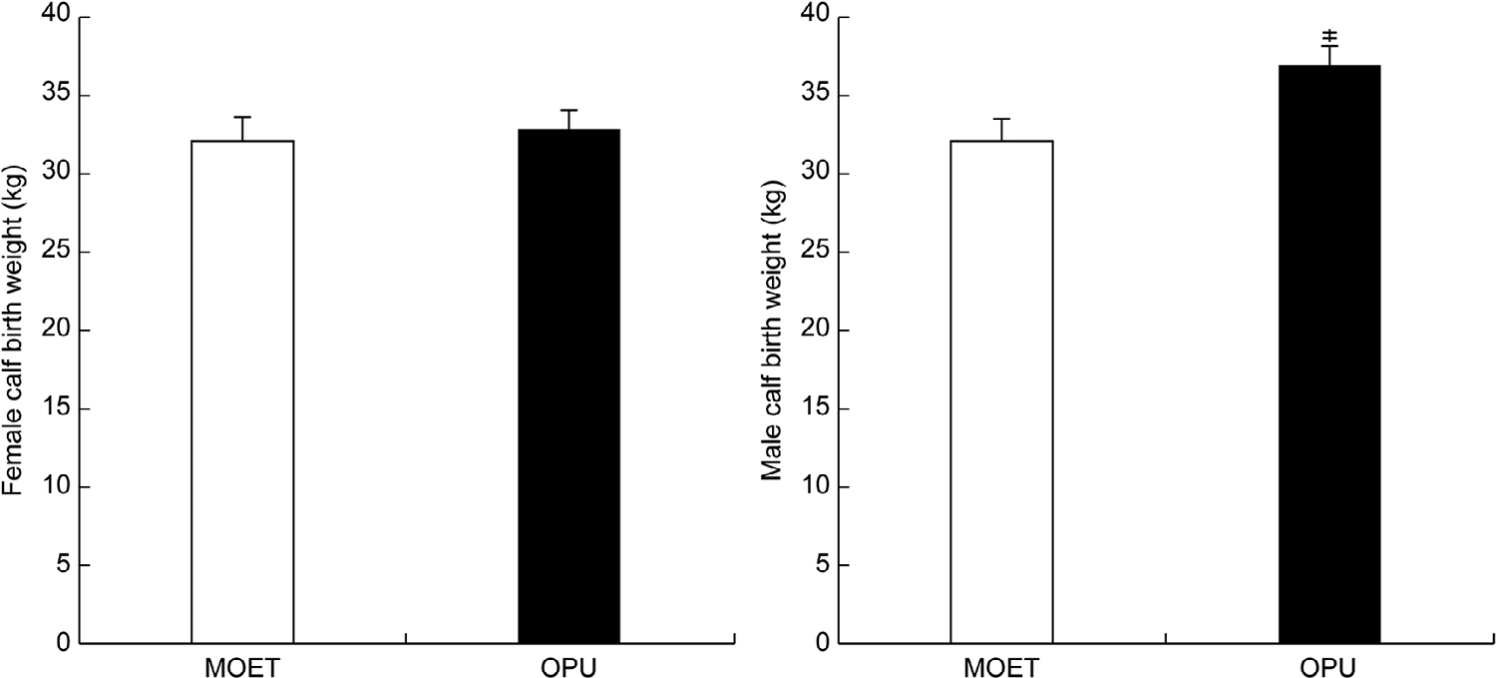
Birth weights of calves in the multiple ovulation and embryo transfer and ovum pick‐up with in vitro fertilization groups. (*) indicates a significant difference (*p* < 0.05).

A comparative analysis of birth weights of female calves between the MOET and OPU embryo groups revealed mean weights of 32.0 ± 1.5 and 32.8 ± 1.3 kg, respectively, with no significant difference. In contrast, the mean weight of male calves was 32.0 ± 1.5 kg in the MOET group and 36.8 ± 1.4 kg in the OPU group (*p* < 0.05) (Figure 2).

### 3.3 Incidence of Dystocia Between the MOET and OPU Groups

**FIGURE 3:**
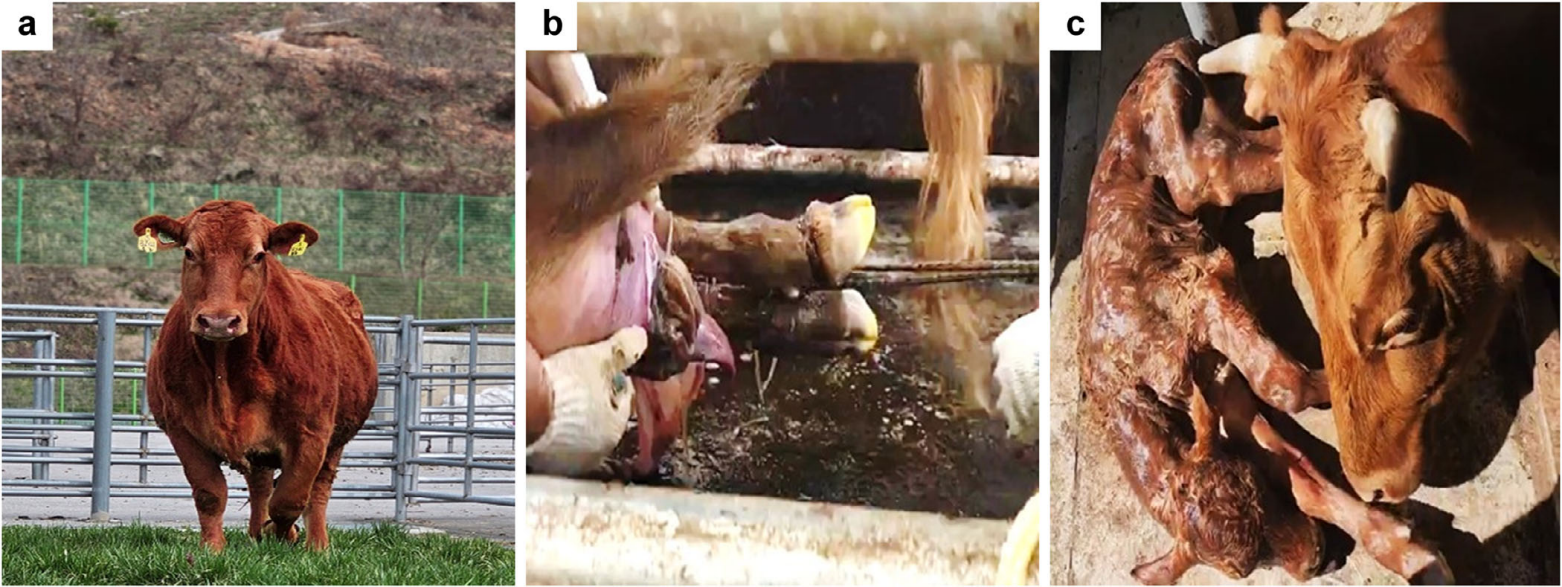
Production of an Elite Hanwoo calf through embryo transfer, delivered via rope‐assisted traction from a surrogate dam experiencing dystocia. (a) Top 0.1% Elite Hanwoo donor cows, (b) rope‐assisted traction for dystocia in recipient Hanwoo cows and (c) elite calf post‐parturition.

Among the 38 recipients that successfully delivered calves, spontaneous parturition occurred in 18 recipients (MOET, 5 out of 8; OPU, 13 out of 30), whereas induced parturition was required in 20 recipients (MOET, 3 out of 8; OPU, 17 out of 30). In the MOET group, the incidence of spontaneous parturition was 62.5%, with 37.5% of cows undergoing induction. In contrast, in the OPU group, spontaneous parturition occurred in 43.3% of cows, whereas induced parturition accounted for 56.7%, indicating a higher rate of labour induction in the OPU group (Table 3, Figure 3).

**TABLE 3:** Pregnancy duration and dystocia incidence according to the calving type of surrogate for embryos derived from Elite Hanwoo cows.

Calving type	MOET (%)	OPU (%)	No. of calving	Gestation period (days)	% Dystocia (head)
Spontaneous delivery	5 (62.5)	13 (43.3)	18	282.9 ± 0.5	11.1 (2)
Induced delivery	3 (37.5)	19 (56.7)	20	286.8 ± 0.3^a^	50.0 (10)

^a^ Indicates a significant difference (*p* < 0.05).

The gestational period differed significantly between the groups. The average gestation length for spontaneous parturition was 282.9 ± 0.5 days and that for induced parturition was 286.8 ± 0.3 days (*p* < 0.05). The incidence of dystocia also varied markedly between the groups. While only 11.1% of the spontaneous parturition cases experienced dystocia, the rate increased significantly to 50.0% in the induced parturition group.

### 3.4 Effect of Timing of Induced Parturition on Calving Outcomes Following Transfer of in Vitro‐Produced Embryos Derived From Elite Hanwoo Cows

Parturition was induced in recipients with in vitro‐produced (IVP) embryos derived from Elite genetic Hanwoo cows. Based on the estimated calving date, it was hypothesized that the foetal volume and birth weight would be larger than the average. Therefore, a single intramuscular injection of a PGF_2_α analogue was administered either 1 day prior (Day −1), on the day of (Day 0) or 1 day after (Day +1) the estimated calving date to induce labour. The assessed outcomes included the rate of daytime calving (for ease of observation), gestational length, calf birth weight and incidence of dystocia (Table 4).

**TABLE 4:** Comparative analysis of the gestation period, daytime delivery rate and average calf weight in induced deliveries in Elite Hanwoo cows.

Treatment day^a^	No. of calving (head)	%Daytime delivery rate^b^	Delivery time (h)	BW (kg)	%Dystocia (head)
Day −1 (277 days)	8	50.0 (4)	55.1 ± 9.8^a^	35.4 ± 1.9	37.5 (3)
Day 0 (278 days)	7	71.4 (5)	24.8 ± 4.6	33.8 ± 1.9	57.1 (3)
Day +1 (279 days)	5	80.0 (4)	35.3 ± 9.8	34.6 ± 1.8	60.0 (4)
Total	20	65.0 (13)	39.5 ± 5.6	34.6 ± 1.1	50.0 (10)

^a^ Induced parturition was conducted based on the estimated calving date (calculated as 278 days for blastocyst transfer, excluding 7 days from the standard 285 days). PGF2α (5 mL) was intramuscularly administered on Days −1, 0, and +1 relative to the estimated calving date (*p* < 0.05).

^b^ Induced deliveries were categorized into daytime deliveries occurring between 7:00 AM and 7:00 PM, and the daytime delivery rate was analysed.

The results showed that recipients that calved on Day −1 delivered calves with an average birth weight of 35.4 ± 1.9 kg. The time from induction to parturition averaged 55.1 ± 9.8 h, and the incidence of dystocia was 37.5% (3/8). In the Day 0 group, calves had an average birth weight of 33.8 ± 1.9 kg, with an average induction‐to‐calving interval of 24.8 ± 4.6 h; dystocia occurred in 57.1% (4/7) of these cases. In the Day +1 group, the average birth weight was 34.6 ± 1.8 kg and time to calving was 35.3 ± 9.8 h; dystocia was observed in 60.0% (3/5) of dams. Across all groups, dystocia occurred in 10 of the 20 animals (50.0%). On the basis of these findings, the high incidence of dystocia could be attributed to the transfer of embryos from Elite Hanwoo cows into standard Hanwoo recipients.

## 4 Discussion

These differences may be explained by epigenetic and developmental disturbances induced during in vitro embryo culture, including altered DNA methylation and imprinting errors, which have been associated with increased pregnancy loss and congenital abnormalities in IVF‐derived embryos compared with in vivo–derived embryos from AI (Young et al. 1998; Farin et al. 2006). The overall rate of miscarriage during pregnancy is also higher in the IVF‐derived ET group than in the AI group. Specifically, the stillbirth rate in the AI group is 7.8%, nearly twice as high as that in the IVF‐derived ET group (14.6%). Calves produced from IVP embryos have a higher incidence of dystocia, higher rate of perinatal mortality and lower survival rate than those produced from AI or in vivo‐fertilized embryos. In addition, contemporary reports indicate that pregnancy and calving outcomes after IVP‐derived ET are substantially influenced by embryo status. Calving rates after fresh IVP‐derived ET have been reported to be comparable to those after AI (approximately 45.5% vs. 44.0%), whereas lower calving rates are observed following frozen IVP‐derived ET (approximately 30.2%) (Lonergan et al. 2025). Moreover, increased pregnancy loss after initial diagnosis in IVP‐ET has been reported in several large‐scale datasets. Therefore, the lower pregnancy and calving rates and the higher miscarriage rates observed in the OPU group compared with the MOET group in the present study are unlikely to be attributable to the genetic quality of elite cattle embryos, but rather to the effects of in vitro culture conditions and the IVF process itself. The overall mean birth weight of calves produced from Elite Hanwoo embryos was 32.4 kg for females and 34.5 kg for males. In comparison, previous reports indicate that the average birth weight of calves of general Hanwoo cows is 28.1 kg for females and 30.4 kg for males (Cho et al. 2021). These findings suggest that calves produced from Elite Hanwoo cow embryos have higher birth weights than those produced from Hanwoo cow embryos.

Dystocia is generally attributed to maternal factors such as poor maternal development and pelvic abnormalities (25%), and foetal factors such as abnormal foetal presentation and macrosomia (75%). Although dystocia can occur even during spontaneous delivery, labour is often induced after the expected due date to ensure the safety of both the dam and foetus, particularly after assessing the maternal condition (Risco et al. 1994).

In the present study, decisions regarding spontaneous versus induced parturition were made based on the clinical assessment of each surrogate cow that received superior Hanwoo‐derived embryos. Among the 38 cows, dystocia occurred in 12 animals (31.6%), with recipients subjected to induced parturition accounting for the majority of cases rather than those undergoing spontaneous delivery. On the basis of these findings, the high incidence of dystocia could be attributed to the transfer of embryos from Elite Hanwoo cows to standard Hanwoo recipients. In previous studies using OPU‐derived embryos from standard Hanwoo cows, the average birth weight was 28.2 kg for females and 30.47 kg for males, with a dystocia incidence of only 3.8% (Cho et al. 2021). The increased birth weight and larger body size of Elite Hanwoo calves likely contribute to a higher risk of calving difficulties. In this study, after the administration of the labour‐inducing agent, parturition occurred within 24.8–55.1 h, with a mean of approximately 39.5 h. A comparison of calf birth weight between dystocia and non‐dystocia cases revealed that calves born with dystocia were significantly heavier, averaging 34.6 kg—approximately 3.5 kg more than those born without complications.

When dystocia was anticipated based on the maternal condition, foetal status was assessed prior to delivery. Calving assistance was provided using obstetric ropes for traction, and in some cases, episiotomy was performed to facilitate delivery and avoid perinatal mortality or placental retention. Generally, when dystocia occurs in cattle, the use of assisted calving techniques, such as controlled traction, as employed in this study, can ensure safe and effective delivery. In severe cases, caesarean section may be indicated (Smail et al. 2025).

## 5 Conclusion

The findings suggest that for pregnancies of Elite Hanwoo cow‐derived ET, induction of labour 1 day prior to the estimated calving date may be a practical strategy to reduce dystocia‐related calf mortality and ensure the successful birth of high‐quality offspring. In addition, close monitoring at the time of parturition and timely intervention may help prevent stillbirths and perinatal deaths, thereby supporting farm productivity and genetic improvement in Hanwoo breeds.

## Author Contributions

Conceptualization: Park MR, Ko YG. Data curation: Lee SY. Formal analysis: Cho SR. Methodology: Lee SY, Ha S. Software: Park MR. Validation: Ha S. Investigation: Park MR, Lee SY, Ko YG. Writing original draft: Park MR, Ko YG. Writing review & editing: Park MR, Lee SY, Cho SR, Ha S, Ko YG. All authors have read and agreed to the published version of the manuscript.

## Conflicts of Interest

The authors declare no conflicts of interest.

## Data Availability

The authors have nothing to report.
